# What Has Neuroimaging Taught Us on the Neurobiology of Yoga? A Review

**DOI:** 10.3389/fnint.2020.00034

**Published:** 2020-07-08

**Authors:** June van Aalst, Jenny Ceccarini, Koen Demyttenaere, Stefan Sunaert, Koen Van Laere

**Affiliations:** ^1^Nuclear Medicine and Molecular Imaging, Department of Imaging and Pathology, UZ/KU Leuven, Leuven, Belgium; ^2^Research Group Psychiatry, Department of Neuroscience, University Psychiatry Center KU Leuven, Leuven, Belgium; ^3^Adult Psychiatry, UZ Leuven, Leuven, Belgium; ^4^Translational MRI, Department of Imaging and Pathology, KU Leuven, Leuven, Belgium; ^5^Department of Radiology, UZ Leuven, Leuven, Belgium; ^6^Division of Nuclear Medicine, UZ Leuven, Leuven, Belgium

**Keywords:** yoga, neuroimaging, PET, SPECT, MRI, neurobiology, connectivity

## Abstract

Yoga is becoming increasingly popular worldwide, with several implicated physical and mental benefits. Here we provide a comprehensive and critical review of the research generated from the existing neuroimaging literature in studies of yoga practitioners. We reviewed 34 international peer-reviewed neuroimaging studies of yoga using magnetic resonance imaging (MRI), positron emission tomography (PET), or single-photon emission computed tomography (SPECT): 11 morphological and 26 functional studies, including three studies that were classified as both morphological and functional. Consistent findings include increased gray matter volume in the insula and hippocampus, increased activation of prefrontal cortical regions, and functional connectivity changes mainly within the default mode network. There is quite some variability in the neuroimaging findings that partially reflects different yoga styles and approaches, as well as sample size limitations. Direct comparator groups such as physical activity are scarcely used so far. Finally, hypotheses on the underlying neurobiology derived from the imaging findings are discussed in the light of the potential beneficial effects of yoga.

## Introduction

The term yoga is derived from a Sanskrit word *yuj* and means “union” or a “method of spiritual union.” Yoga originated in India and is a behavioral practice that unites physical and mental training aimed at improving health and promoting personal transformation, with the ultimate goal of attaining *samadhi* (pure consciousness). According to *Patanjali*’s *Sutras*, yoga encompasses the following eight aspects (limbs): *yamas* (ethical guidelines, abstinence from immoral behavior), *niyamas* (self-discipline), *asana* (physical postures), *pranayama* (breath control), *pratyahara* (sensory withdrawal), *dharana* (concentration), *dhyana* (meditation), and *samadhi* (pure consciousness) (Villemure et al., 2014, 2015; Birdee et al., 2016; Eyre et al., 2016; Khalsa et al., 2016). Over the years, dozens of variations in yoga philosophy and styles have emerged. Some yoga styles are structured as a physical workout, while others put an emphasis on meditation. Meditation is a way of focusing and slowing down the stream of thoughts in the mind (Brewer et al., 2011). *Dhyana*, Sanskrit for meditation, is one of the eight limbs of yoga according to *Patanjali’s Sutras* and stresses the fact that yoga and meditation are interrelated. Several meditation techniques originate from yoga and are intrinsically connected with yoga. In addition, to train the mind to focus and prepare for meditation, physical movements of the body and breathing exercises are used and together from the triad of yoga trainings (Birdee et al., 2016).

Whereas yoga has been practiced in the East for thousands of years, it is now rapidly gaining popularity and interest in the Western world (Ivtzan and Jegatheeswaran, 2015). Data from a recent national health interview survey showed that 14.3% of the United States adults have done yoga in the past 12 months (Clarke et al., 2018). Furthermore, yoga practitioners are more likely female, younger, non-Hispanic white, college educated, higher earners, and of better health status (Cramer et al., 2016). The goal for many healthy individuals is to achieve fitness and flexibility, to reduce daily stress and improve energy as part of a healthy, active lifestyle (Barnes et al., 2008; Ivtzan and Jegatheeswaran, 2015; Cramer et al., 2016). Several yoga styles are practiced in western society, and most encompass the abovementioned triad of physical postures, breath control exercises, and meditation, although in many centers the primary focus is put on the *asana* (physical postures) of yoga (Ivtzan and Jegatheeswaran, 2015). There are also different intentions to practice yoga that may vary from purely physical to more spiritual. A recent study showed that the motivations to practice yoga are dynamic. With continued practice, there is a shift in intentions that are set by practitioners from more physical to spiritual development (Ivtzan and Jegatheeswaran, 2015).

On the other hand, yoga is also regularly practiced by people with physical and mental health problems. Cramer et al. (2016) performed an analysis on the cross-sectional data from the 2012 National Health Interview Survey. In this survey, most yoga practitioners reported positive outcomes resulting from their yoga practice, predominantly citing reduced stress, improved overall health, improved emotional well-being, improved sleep, and increased sense of control over their health. In addition to these subjective self-reported benefits, there is a growing body of clinical research studies on beneficial medical and psychological effects of yoga. Physiological effects of yoga include a decreased heart rate and blood pressure, and increased muscle strength (Hagins et al., 2014; Vardar Yağlı et al., 2015; Chu et al., 2016). Furthermore, improvement of depressive, anxious, and stressful states and relief in pain conditions has been demonstrated in several studies (Woolery et al., 2004; Cramer et al., 2013; Danucalov et al., 2013; Riley and Park, 2015; Wieland et al., 2017). Yoga may thus also offer a complementary auxiliary approach in various central nervous system (CNS) disorders such as depression, anxiety, posttraumatic stress disorder (PTSD) but also in schizophrenia and cognitive decline (Vancampfort et al., 2012; Cramer et al., 2013; Riley and Park, 2015; Brenes et al., 2018). Mostly, these effects are measured with self-reported questionnaires before, during, and after a yoga intervention and such an approach may be prone to bias and subjectivity. Non-invasive and easily accessible (bio)markers, for example blood pressure, medication use, and heart rate (variability), have been used as measurements of interest but remain aspecific.

There is growing interest in elucidating the neurobiological underpinnings of yoga. There are different methods to non-invasively investigate the neurobiology of the human brain including magnetic resonance imaging (MRI), positron emission tomography (PET), single-photon emission computed tomography (SPECT), electroencephalography (EEG), magnetoencephalography (MEG), transcranial direct-current stimulation (TDCS), transcranial magnetic stimulation (TMS), and blood biomarker analysis. For this review, our focus is on neuroimaging studies using MR-based techniques, PET, and/or SPECT, since these non-invasive modalities allow to to investigate the brain’s structure and function *in vivo* and network functional effects *in vivo* with high spatial resolution (MRI) and sensitivity (PET).

The main purpose of the present review is to provide an overview of the existing structural and functional neuroimaging studies of yoga in a critical perspective, by examining the magnitude and the consistency of reported cerebral effects. We will address inconsistencies, highlight the gaps in our current knowledge, and discuss how better study designs and imaging probes may be able to address these.

## *In vivo* Brain Imaging Techniques

MRI, PET, and SPECT have been the medical neuroimaging techniques of choice to investigate the effects of yoga on the brain.

MRI refers to a family of imaging techniques that use strong magnetic fields, field gradients, and radio waves to excite hydrogen atoms in tissues and read out signals, depending on the properties of these tissues (Hendee and Morgan, 1984). The main advantages of MRI are its (sub)millimeter spatial resolution, non-invasiveness, and ability to discriminate tissues using their physical and biochemical properties. On the other hand, a relatively low sensitivity is a main disadvantage (Catana et al., 2013). MRI can be used to assess both cerebral structural and functional effects. Structural MRI (sMRI) provides anatomical information, delineating different tissues and brain structures, and can be used to assess volumetric changes or differences between populations. To assess and investigate possible changes in the white matter (WM) of the brain, diffusion tensor imaging (DTI) is commonly used (Sasson et al., 2010). DTI is sensitive to the diffusion characteristics of hydrogen. When diffusion is not restricted, hydrogen diffuses the same amount in all directions; the diffusion is called “isotropic.” In contrast, when hydrogen diffusion is restricted and dominated by one direction, the diffusion is called “anisotropic.” In WM, diffusion tends to be anisotropic due to the axon walls and myelin sheaths surrounding the axon, which is picked up in DTI, which is thus well-suited to investigate WM tract integrity. In addition to sMRI, functional MRI (fMRI) measures can be acquired, including resting-state or task fMRI, functional connectivity fMRI, magnetic resonance spectroscopy (MRS), and arterial spin labeling (ASL). *In vivo* MRS is a non-invasive specialized technique associated with MRI, where the differences in resonant frequency of various chemical compounds are measured and observed, in order to identify the MRS spectrum. This MRS spectrum reflects the biochemical composition of a region of interest (or whole brain by voxel-based measurements), and each metabolite is identified by its unique position (Henning, 2018): GABA, glutamate + glutamine (Glx), reflecting major inhibitory and excitatory neurotransmitters, glycerophosphocholine + phosphocholine (Cho), a marker of cell membrane syntheses and breakdown, and N-acetyl aspartate and N-acetylaspartyl-glutamate (NAA/NAAG), a marker of neuronal and axonal viability and density (Verma et al., 2016). In resting-state or task fMRI, regional brain activity is detected based on blood oxygenation level-dependent (BOLD) changes in brain tissue that change with neuronal activity status (Chen and Glover, 2015). Correlation patterns of the activated regions can be identified with a functional connectivity analysis. Several networks have been robustly identified, including the default mode network (DMN), which is active in the absence of an attention-required task (Fox and Greicius, 2010), and the central executive (frontal) network (Trotta et al., 2018). Indirect neuronal functioning can be measured using ASL measuring absolute cerebral perfusion. In this technique, incoming arterial blood is magnetically labeled and the signal difference between labeled and unlabeled control images is proportional to regional perfusion (Haller et al., 2016).

PET is a molecular functional nuclear medicine imaging technique with very high (nanomolar) sensitivity. PET imaging makes use of a radioactively labeled radioligand administered in tracer quantities. Radiotracers consisting of a positron-emitting radioistope are bound to a compound that binds to target molecules such as neuroreceptors, reuptake transporters, or intracellular and extracellular proteins. Positron emission is located through measurement of coincident annihilation gamma rays detected by scintillator or solid-state PET-scan detectors (Sossi, 2018), and a quantitative physiological measure can be obtained such as receptor density or glucose metabolization rate. Although spatial resolution is less than that of sMRI, current equipment allows 3–4 mm spatial resolution (Vandenberghe et al., 2016).

Similarly, SPECT imaging is also used to visualize molecular and functional brain processes. In contrast to PET, the tracers are labeled with a single-photon emitting radioisotope. SPECT imaging provides quantitative images of the radiotracer distribution using a rotating scintillator gamma camera. In functional brain studies, SPECT has mainly been used to study regional brain perfusion (Goffin and van Laere, 2016).

## Review Method and Search Strategy

Articles were identified using MEDLINE^1^ until the end of 2019, limiting the search to yoga and neuroimaging studies, using the following keywords: “yoga AND (magnetic resonance OR positron emission tomography OR single photon emission computed tomography OR MR OR PET OR SPECT or functional connectivity).” The obtained list of articles was afterward refined and screened for inclusion of at least one of the following words in the abstract: “magnetic resonance OR MR,” “functional magnetic resonance imaging OR fMRI,” “positron emission tomography OR PET,” “single photon emission computed tomography OR SPECT,” or “brain” AND “yoga” within the title or abstract (see below the study inclusion and exclusion criteria). Of the remaining results, all the abstracts were verified to describe neuroimaging methods in the investigation of cerebral effects of yoga.

We considered all studies using structural and functional neuroimaging to investigate different forms of yoga styles that met the following inclusion criteria: (i) articles that were available in English, German, Dutch, or French; (ii) articles that used neuroimaging techniques; and (iii) articles published in peer-reviewed scientific journals.

Studies were excluded in case of (i) individual case reports; (ii) effects of one-time practitioners; (iii) review articles; (iv) using neurophysiological techniques other than MRI, PET, or SPECT (e.g., EEG mapping, MEG, etc.).

## Results

### Study Selection and Classification

A total of 93 studies were identified and reviewed. About two-thirds (59 of 93) of all studies did not meet the inclusion criteria, mainly because these studies did not focus on yoga itself or did not investigate neuronal effects, ending with a final set of 34 studies.

The included studies used different styles of yoga, which are briefly outlined in the Supplementary Appendix: Ashtanga, Iyengar, Vinyasa, Kripalu, Kundalini, Nidra, Sahaja, Sivananda, and Hatha yoga. The selected 34 articles were straightforward classified as “*structural*/*morphological neuroimaging studies*,” which assessed structural brain changes by sMRI (*n* = 11, Table 1), or “*functional neuroimaging studies*,” which assessed brain function, activation studies, or molecular targets (*n* = 26 studies, Table 2). The latter comprised MRS, task-based fMRI, resting-state fMRI (rsfMRI) with connectivity analysis, ASL, PET, and SPECT. Three of the 34 included articles used a combination of structural and functional imaging and were included in both sections. Six pairs of articles conducted different analyses in the same subjects and were included in both the structural and functional study sections.

**TABLE 1:** Morphological brain imaging studies on yoga.

Imaging technique	Effect	Design	Yoga style	Number (♀), age (mean ± SD), yrs	Control group Control condition	Study
MRI-VBM	WB GMD	CSS	Hatha Yoga meditation (HYM) (MY)	HYM: 7 (6), 36.4 ± 11.9 CON: 7 (6), 35.5 ± 7.1	Non-meditators vs. yoga	Froeliger et al., 2012b
MRI-VBM MRI-VBCT MRI-DTI	WB GMD, GMT, ROI WM	CSS	Unspecified yoga (3Y)	YOG: 14 (9), 37.0 ± 6.6 CON: 14 (9), 36.7 ± 7.3	Non-meditators vs. yoga	Villemure et al., 2014
MRI-VBM	WB GMD	CSS	Unspecified yoga (3Y)	YOG: 14 (9) 37.0 ± 6.6 CON: 14 (9) 36.7 ± 7.3	Non-meditators vs. yoga	Villemure et al., 2015
MRI-VBM	WB GMD	CSS	Sahaja Yoga meditation (SYM)(YM)	SYM: 23 (17), 46.5 ± 11.4 CON: 23 (17), 46.9 ± 10.9	Non-meditators vs. yoga	Hernández et al., 2016
MRI-VBM	WB GMD	LS	Sahaja yoga meditation (SYM)(MY)	SYM: 12 (2): 21.6 ± 2.0 CON: 30 (12): 22.2 ± 1.3	SYM training vs. control group (waiting period)	Dodich et al., 2019
MRI-VBCT	WB GMT	CSS	Hatha Yoga (3Y)	YOG: 21 (21), 66.2 ± 4.5 CON: 21 (21), 67.9 ± 4.6	Non-meditators vs. yoga	Afonso et al., 2017
MRI-VBM	ROI GMV	CSS LS	Meditation and yoga practices (MY/3Y)	YOG: 289 (211), 61.9 ± 6.8 CON: 3453 (1830), 64.3 ± 7.7	Controls vs. practitioners of yoga, meditation and breathing exercises	Gotink et al., 2018
MRI-VBM	ROI GMD	LS	Yoga (3Y)	YOG: 7 (3), 69–81*	Before vs. after yoga training	Hariprasad et al., 2013
MRI-VBM	ROI GMD	CSS	Hatha, iyengar and kundalini yoga (3Y)	YOG: 13 (12), 35.8 ± 15.4 CON: 13 (12), 35.7 ± 14.6	Controls vs. yoga practitioners	Gothe et al., 2018
MRI-VBM	ROI GMD	LS	MBSR program (including yoga) (MY/3Y)	YOG: 27 (16), 35.2 ± 6.7	Before vs. after MBSR training	Hölzel et al., 2009
MRI-VBM	ROI GMV	LS	Combination of meditation and Kundalini yoga (MY/3Y)	YOG: 14 (6), 67.1 ± 9.5 CON: 11 (6), 67.8 ± 9.7	MET vs. yoga in MCI patients	Yang et al., 2016

Imaging Technique: DTI, diffusion tensor imaging; MRI, magnetic resonance imaging; VBM, voxel-based morphometry; VBCT, voxel-based cortical thickness. Effect: GMD, gray matter density; GMV, gray matter volume; GMT, gray matter thickness; GM, gray matter; ROI, region of interest; WB, whole-brain; WM, white matter. Design: CSS, cross-sectional study; LS, longitudinal study. Yoga styles: 3Y, triad of yoga (including physical postures, breathing exercises, and meditation); HYM, Hatha Yoga meditation; MBSR, mindfulness-based stress reduction; MY, meditation yoga; SYM, Sahaja Yoga meditation. Number: SD, standard deviation. Control group Control condition: MCI, mild cognitive impairment; MET, memory enhancement training. * No information on mean age and SD.

**TABLE 2:** Functional brain imaging studies on yoga.

Imaging technique	Effect	Design	Yoga style	Number (♀), age (mean ± SD), yrs	Control group Control condition	Study
**A. Cerebral perfusion and glucose metabolism**						
^18^F-FDG PET	Glucose metabolism	CSS	Yoga meditation (YM)	YOG: 8 (2), 32, 21–39*	Wakeful condition in the same subjects	Herzog et al., 1991
H_2_^15^O PET	CBF	CSS	Yoga Nidra (YM)	YOG: 9 (3), 23–41*	Control states in the same subjects	Lou et al., 1999
^99m^Tc-ECD SPECT	CBF	LS	Iyengar yoga (3Y)	YOG: 4 (2), 45.0	Pre-program baseline scan	Cohen et al., 2009
^99m^Tc-HMPAO SPECT	CBF	CSS	Kundalini chanting (YM)	YOG: 11 (5), 35.4 ± 13.5	BL in same subjects	Khalsa et al., 2009
fMRI ASL	CBF	CSS	Kundalini meditation (YM)	YOG: 10 (4), 53.7*	BL in same subjects	Wang et al., 2011
**B. Neural activation**						
fMRI	Neural activation	CSS	Kundalini, Acem tradition (MY)	YOG: 8 (5), 34.6 ± 9.7	BL in same subjects	Engström et al., 2010
fMRI	Neural activation	CSS	OM chanting (MY)	YOG: 12 (3), 28 ± 6	Production of “ssss…”	Kalyani et al., 2011
fMRI	Neural activation	CSS	Sahaja yoga meditation (MY)	YOG: 19 (11), 46.6 ± 9.5	Attention on breathing	Hernández et al., 2015
fMRI	Neural activation	CSS	*Patanljali* yoga: Yoga meditation (MY)	YOG: 4 (0), mid 60s*	Relaxation (control condition) vs. meditation	Mishra et al., 2017
fMRI	Neuronal fluctuations	LS	Hatha Yoga (3Y)	CON: 12 (12), 16–60* YOG: 23 (23), 16–60* AE: 23 (23), 16–60*	Patients with schizophrenia: control wait-list vs. yoga vs. aerobic exercises (AE)	Lin et al., 2017
fMRI	Neural activation	CSS	Undefined	YOG: 19 (16), 35.9 ± 11.5 CON: 12 (6), 32.9 ± 9.1	Recreational athletes vs. yoga practitioners (YP)	Wadden et al., 2018
fMRI	Neural activation	CSS	Hatha meditation (MY)	YOG: 7 (6), 36.4 ± 11.9 CON: 7 (6), 35.5 ± 7.1	Meditation-naive vs. controls	Froeliger et al., 2012c
fMRI	Neural activation	CSS	Hatha, iyengar, and kundalini (MY/3Y)	YOG: 13 (12), 35.8 ± 15.4 CON: 13 (12), 35.7 ± 14.6	Controls vs. yoga practitioners	Gothe et al., 2018
**C. Functional connectivity**						
fMRI	Functional connectivity	CSS	Hatha meditation (MY)	YOG: 7 (6), 36.4 ± 11.9 CON: 7 (6), 35.5 ± 7.1	Meditation-naive vs. controls	Froeliger et al., 2012a
fMRI	Functional connectivity	CSS	Sahaja yoga meditation (SYM)(YM)	SYM: 23 (17), 46.5 ± 11.4 CON: 23 (17), 46.9 ± 10.9	Meditation state vs. resting state	Hernández et al., 2018
fMRI	Functional connectivity	LS	MBSR program (including yoga) (MY/3Y)	MBSR: 18 (10): 37.5 ± 9.1 RR: 16 (9): 39.9 ± 10.3	MBSR vs. relaxation response RR training	Sevinc et al., 2018
fMRI	Functional connectivity	CSS	Hatha yoga (3Y)	CON: 20 (20), 68.2 ± 4.6 YOG: 20 (20), 66.5 ± 4.5	Elderly yoga practitioners vs. healthy yoga-naïve controls	Santaella et al., 2019
fMRI	Functional connectivity	LS	Meditation/Kundalini yoga (MY/3Y)	YOG: 14 (6), 67.1 ± 9.5 MET: 11 (6), 67.8 ± 9.7	MET vs. yoga in MCI patients	Eyre et al., 2016
fMRI	Functional connectivity	LS	Sahaja yoga meditation (SYM)(MY)	SYM: 12 (2): 21.6 ± 2.0 CON: 30 (12): 22.2 ± 1.3	SYM training vs. control group (waiting period)	Dodich et al., 2019
fMRI	Functional connectivity	CSS	Kripalu yoga, Vipassana meditation (MY/3Y)	YOG: 16 (11), 49.4 ± 7.8 MED: 16 (10), 54.1 ± 8.1 CON: 15 (9), 52.9 ± 9.8	Meditation vs. yoga vs. controls	Gard et al., 2014
**D. Brain metabolites and neurotransmitters**						
^11^C-raclopride PET	Dopamine release	CSS	Yoga Nidra (MY)	YOG: 8 (0), 31–50*	Wakeful condition in same subjects	Kjaer et al., 2002
MRI-MRS	GABA	CSS	Yoga (3Y)	YOG: 8 (7), 25.8 ± 5.2 CON: 11 (5), 26.6 ± 7.6	Reading exercise	Streeter et al., 2007
MRI-MRS	GABA	LS	Iyengar yoga (3Y)	YOG: 19 (11), 23.9 ± 3.0 CON: 15 (11) 25.6 ± 4.9	Walking group	Streeter et al., 2010
MRI-MRS	NAA and MI	CSS	Yoga postures and breathing exercises (3Y)	YOG: 34 (?), 35–65* CON: 34 (?), 35–65*	Type 2 diabetes patients: Yoga + standard care vs. standard care	Nagothu et al., 2015
MRI-MRS	NAA and MI	CSS	Yoga postures and breathing exercises (3Y)	YOG: 5 (?), 35–55* CON: 5 (?), 35–55*	Type 2 diabetes patients: Yoga + standard care vs. standard care	Santhakumari et al., 2016
MRI-MRS	Metabolites	LS	Meditation/Kundalini yoga (MY/3Y)	YOG: 14 (6), 67.1 ± 9.5 CON: 11 (6), 67.8 ± 9.7	MET vs. yoga in MCI patients	Yang et al., 2016

Imaging technique: ASL, arterial spin labeling; FDG, fluorodeoxyglucose; (f)MRI, (functional) magnetic resonance imaging; MRS, magnetic resonance spectroscopy; PET, positron emission tomography; SPECT, single-photon emission computed tomography. Effect: CBF, cerebral blood flow; GABA, γ-aminobutyric acid; MI, myoinositol; NAA, N-acetyl aspartate. Design: CSS, cross-sectional study; LS, longitudinal study. Yoga styles: 3Y, triad of yoga (including physical postures, breathing exercises and meditation); MBSR, mindfulness-based stress reduction; MY, meditation yoga. Number: SD, standard deviation. Control group/control condition: MET, memory enhancement training. * No information on mean age and SD.

### Structural/Morphological Neuroimaging Studies on Yoga

All structural studies carried out in yoga practitioners between 2009 and 2019 performed voxel-based morphometry (VBM) analysis. VBM is a technique to quantitatively assess gray matter (GM) volume in predefined regions of interest (ROIs) or concentration (density) differences throughout the brain on a voxel-by-voxel or ROI basis (Hutton et al., 2009). Additionally, a voxel-based cortical thickness (VBCT) analysis can be performed, where cortical thickness is assessed by calculating the distance between the resulting WM-GM surface and GM-cerebral spinal fluid surface data (Lüsebrink et al., 2013).

Several studies investigated morphological effects conducting a whole-brain analysis without *a priori* restriction to particular ROIs. Froeliger et al. (2012b) found higher GM density in a small group of experienced Hatha yoga meditation practitioners (*n* = 7) compared to a sex-, age-, and education-matched control group (*n* = 7). Particularly, higher GM density was found in the medial frontal gyrus, superior frontal gyrus, precentral gyrus, (para)hippocampal gyrus, insula, superior temporal gyrus, occipital gyrus, and cerebellum (Froeliger et al., 2012b). Similarly, greater GM density in cortical regions were also identified by Villemure et al. (2014), conducted in a group of experienced yoga practitioners (*n* = 14) vs. physically active controls (*n* = 14; matched in terms of sex, age, body mass index, handedness, education, and exercise level outside of yoga). These regions comprised cingulate gyrus, superior frontal gyrus, inferior parietal lobule, and insula. In addition, VBCT showed three brain regions with significantly increased GM thickness in yoga participants, including cingulate cortex, insular cortex, and primary somatosensory cortex, in agreement with the VBM analysis (Villemure et al., 2014). In a follow-up study in the same subjects, the authors also explored whether a yoga practice had a neuroprotective effect by comparing age-related GM decline (Villemure et al., 2015). They found a significant negative correlation between age and whole-brain GM density for controls, consistent with known atrophy effects of healthy aging, but this decline was not present in yoga practitioners. Furthermore, the years of yoga experience and hours of weekly yoga practice were positively correlated with regional GM density (Villemure et al., 2015). In experienced Sahaja yoga meditation participants (*n* = 23) compared to controls (*n* = 23), significantly larger GM density was found in the insula, ventromedial orbitofrontal cortex, and medial inferior temporal gyrus (Hernández et al., 2016). In subjects practicing the same yoga style, Dodich et al. (2019) investigated the modulation of a short-term yoga meditation training (four 1-hour sessions per week over four consecutive weeks), by randomly assigning 42 healthy meditation-naïve adults to either a control group (wait-list; *n* = 30) or a yoga group (*n* = 12). Compared to the controls, the yoga group showed increased GM density in the inferior frontal gyrus (pars orbitalis), correlated with general well-being after yoga training. Finally, in another study, Afonso et al. (2017) found increased cortical thickness in the left prefrontal area in 21 healthy elderly female experienced (practicing at least two times a week for a minimum of eight years) Hatha yoga practitioners compared to 21 age-, education-, and physical activity-matched controls.

Based on defined *a priori* hypotheses on behavioral effects of yoga such as reduction of anxiety and increased attention and their functional anatomical relationship, other studies analyzed structural MR data focusing on effects within specific ROIs, including the hippocampus, amygdala, or dorsal anterior cingulate cortex. Gotink et al. (2018) investigated the effect of meditation and yoga on the amygdala (as a central relay structure in emotional memory, fear, and anxiety processing) and hippocampus. In this large population-based study, including 3742 participants, of whom 289 practiced at least one hour of meditation or yoga per week for at least one year, a decrease in GM volume was observed in the left hippocampus and right amygdala (Gotink et al., 2018). It is worth mentioning that no distinction was made between yoga and meditation practice in this study, therefore obscuring the exact origin of the observed effects. The same study also described an additional analysis in a subsample of 218 subjects that had undergone a previous MR scan five years earlier and have been practicing yoga or meditation for five years or longer. In this longitudinal study, only an interaction between years of yoga/meditation experience and decreased volume of the amygdala was observed, without differences in the hippocampus (Gotink et al., 2018). Similar results were found in a small yoga intervention study (including physical postures, breath control exercises, and meditation) using VBM, performed in healthy elderly subjects (*n* = 7), without a control group (Hariprasad et al., 2013). GM density changes in the hippocampus as *a priori* ROI were examined after the yoga intervention of six months. The hippocampus is known to be affected by GM loss with aging, and previously elevations in serum brain-derived neurotrophic factor (BDNF) had been demonstrated in this region. Increased GM density in the hippocampus post-yoga intervention was found compared to baseline (Hariprasad et al., 2013). Based on the former results, Gothe et al. (2018) explored differences in GM density of the hippocampus specifically in 13 experienced yoga practitioners and 13 age- and sex-matched controls. The experienced yoga group contained Hatha, Kundalini, and Iyengar yoga practitioners. GM density was higher in the left hippocampus in the yoga group compared to controls (Gothe et al., 2018). Other structural imaging research focused on both the hippocampus and amygdala in order to investigate the effects of an 8-week mindfulness-based stress reduction (MBSR) intervention in subjects with elevated stress (*n* = 26), consisting of weekly group meetings and daily home mindfulness practices, including sitting meditation and yoga (Hölzel et al., 2009). Differences in the perceived stress scale were related to changes in GM density within the right amygdala (Hölzel et al., 2009). Finally, in a particular study, aiming at investigating neuroanatomical and neurochemical plasticity following either memory training or a yoga intervention in mild cognitive impairment (MCI) patients, Yang et al. (2016) conducted a longitudinal MR study to measure GM volume within the bilateral hippocampus and dorsal anterior cingulate over 12 weeks. In total, 25 subjects were included and randomized to either the yoga group (*n* = 14) or the control group [memory enhancement training group (*n* = 11)]. An interaction between group and time was found: only the MCI patients who followed memory training sessions showed a trend toward increased volume in the dorsal anterior cingulate cortex after training (Yang et al., 2016).

Only one study has investigated the effects of yoga on WM integrity. In this study, Villemure et al. (2014) performed an ROI-based DTI analysis of the insular cortex in an experienced yoga cohort (*n* = 14) compared to controls (*n* = 14). They found higher fractional anisotropy (FA) in WM adjacent to the left insula compared to controls. Probabilistic tractography was used to track the WM pathway passing through this area, showing increased connectivity of the anterior and posterior insular regions. According to the authors, this might represent increased intrainsular connectivity in the yoga subjects (Villemure et al., 2014).

**FIGURE 1:**
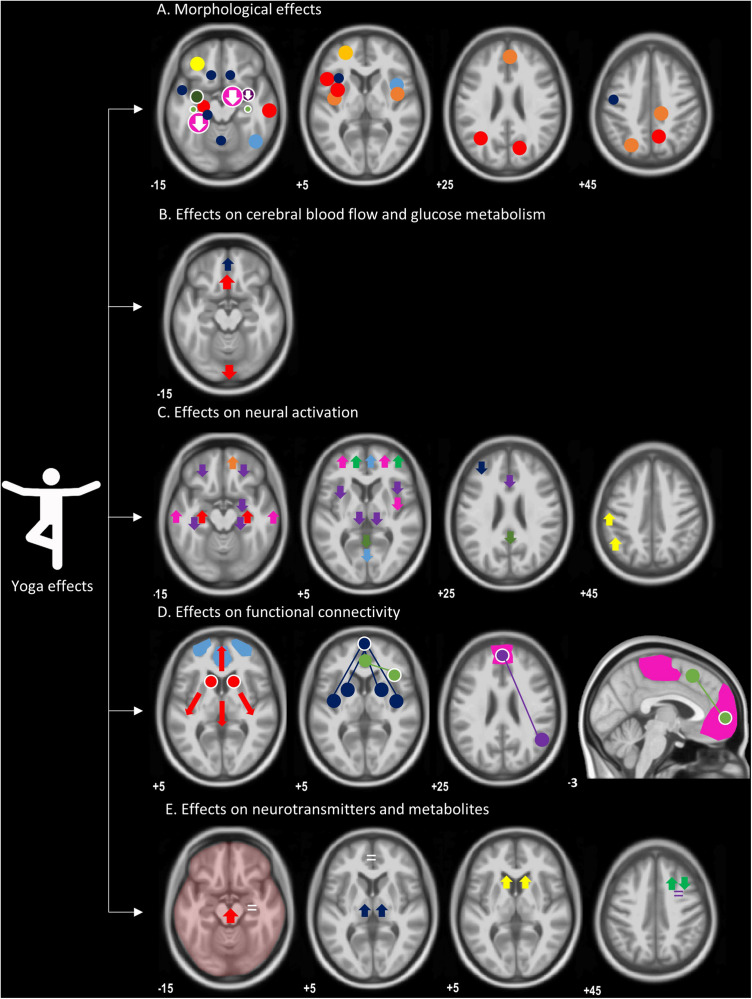
An overview of morphological and functional effects of yoga, overlaid on a T1-weighted image. **(A)** Morphological effects: circles indicate regions with increased gray matter volume or density; regions with a decrease are represented by an arrow pointing down. Both whole-brain and ROI analyses are included, represented by circles without or with a white border, respectively. Size of the circles are a representation of group sizes: *light blue* (Hernández et al., 2016), *dark blue* (Froeliger et al., 2012b), *orange* (Villemure et al., 2014), *red* (Villemure et al., 2015), *yellow* (Dodich et al., 2019), *dark yellow* (Afonso et al., 2017), *pink* (Gotink et al., 2018), *dark green* (Gothe et al., 2018), *light green* (Hariprasad et al., 2013), *purple* (Hölzel et al., 2009), not in this figure (Yang et al., 2016). **(B)** Neuronal resting-state function: *red* (Herzog et al., 1991), *blue* (Khalsa et al., 2009), not in this figure (Lou et al., 1999; Cohen et al., 2009; Wang et al., 2011). **(C)** Neural activation: *red* (Engström et al., 2010), *purple* (Kalyani et al., 2011), *green* (Froeliger et al., 2012c), *pink* (Hernández et al., 2015), *orange* (Mishra et al., 2017), *yellow* (Wadden et al., 2018), *dark blue* (Gothe et al., 2018), and *light blue* (Lin et al., 2015). **(D)** Functional connectivity, seed regions (circles with a white border) connected (full lines) with brain regions (circles), representing higher functional connectivity: *red* (Gard et al., 2015); higher degree centrality for caudate nuclei, *blue* (Hernández et al., 2018), *green* (Sevinc et al., 2018), *pink* (Eyre et al., 2016), *purple* (Santaella et al., 2019), *light blue* represents a network with a change in power spectrum (Dodich et al., 2019), not in this figure (Froeliger et al., 2012a). **(E)** Neurotransmitters and metabolites: *yellow*, increased dopamine signaling (Kjaer et al., 2002); *red*, increased GABA without exact location (Streeter et al., 2007); *dark blue* (GABA levels) (Streeter et al., 2010); *green*, increased N-acetyl-aspartate (NAA) and decrease myoinositol (MI) (Nagothu et al., 2015); *purple*, no change in MI or NAA (Santhakumari et al., 2016); *white*, no change in metabolite levels in hippocampus and dorsal anterior cingulate cortex (Yang et al., 2016).

We have pooled the study specifics of the morphological studies in Table 1. Furthermore, Figure 1A shows a map of the different brain regions where morphological effects of yoga have been reported in the included studies. The insular cortex and hippocampus were the most frequently described regions with morphological thickening.

### Functional Neuroimaging Studies on Yoga

Twenty-six studies have addressed the functional effects of yoga with neuroimaging, of which 21 studies used MR-based techniques, three studies used PET (including assessments of glucose metabolism—with ^18^F-fluorodeoxyglucose (^18^F-FDG), cerebral blood flow or perfusion—with ^15^O-H_2_O, and dopaminergic functioning—with ^11^C-raclopride), and the two remaining studies used SPECT perfusion. We have grouped these studies in four coherent domains: effects on (mainly) resting-state function (perfusion and glucose metabolism), brain activation studies, network function (connectivity analyses), and molecular changes (MRS and neurotransmitters).

#### Effects of Yoga on Cerebral Perfusion and Glucose Metabolism

Changes in neuronal resting-state function in yoga subjects have been investigated in one glucose-metabolism PET study, one perfusion PET study, two perfusion SPECT studies, and one perfusion ASL MR study (Table 2A and Figure 1B).

Herzog et al. (1991) performed historically the first PET imaging study in yoga subjects, aiming to find possible alterations of cerebral glucose metabolism. A group of eight experienced healthy yoga meditation volunteers were scanned with ^18^F-FDG PET in a 2-day protocol, during a normal wakeful resting control state and a yoga meditative relaxation state. Absolute regional cerebral metabolic rates of glucose (rCMRGlc) were obtained with dynamic ^18^F-FDG PET imaging and arterial blood sampling, but no significant differences were found. Several significant regional differences of rCMRGlc ratios were found with increased glucose metabolism in the prefrontal to occipital ROIs, prefrontal to occipitotemporal ROIs, and superior frontal to superior parietal ROI ratio. Furthermore, during the state of yoga meditative relaxation, a smaller intersubject coefficient of variation was observed (Herzog et al., 1991).

In the first cerebral perfusion study, Lou et al. (1999) examined the neural regions subserving Nidra yoga meditation in nine experienced subjects using ^15^O-H_2_O PET. Subjects were injected eight times with ^15^O-H_2_O: two during an normal resting state, two during a resting state with auditory stimulation, and four during meditation, which was induced and maintained by auditory stimulation. While the global cerebral blood flow (CBF) remained unchanged throughout the experiment, a regional altered CBF pattern of meditation was observed according to the meditative content. Additionally, differential regional CBF activity upon meditation was found in regions thought to support an executive attentional network, such as the orbital and dorsolateral prefrontal gyrus, anterior cingulate gyrus, temporal gyrus, pons, and cerebellum (Lou et al., 1999). With the use of ^99m^Tc-ECD (ethylcysteine dimer or bicisate) SPECT, perfusion was also examined in a small longitudinal, interventional study where participants (*n* = 4) underwent a 12-week Iyengar yoga training program (Cohen et al., 2009). Each participant received both a pre-program baseline and meditation scan and a post-program baseline and meditation scan. A significant decrease between the mean CBF ratios in the pre- and post-program baseline scans was detected in the following ROIs: amygdala, dorsal medial frontal cortex, and precentral and postcentral gyrus. Significant differences in pre- and post-program percentage change were observed in the medial frontal gyrus, prefrontal cortex (PFC), precentral gyrus, postcentral gyrus, and inferior and superior frontal gyrus (Cohen et al., 2009). ^99m^Tc-HMPAO (hexamethylpropylene amine oxime or exametazime) SPECT was performed to examine perfusion changes during a Kundalini yoga chanting meditation in 11 experienced meditation participants (Khalsa et al., 2009). This study showed decreased rCBF during meditation compared to the control condition in the middle occipital, superior parietal, inferior temporal, and medial frontal gyrus. In contrast, during the meditation an activation was found in the posterior cingulate gyrus and left superior temporal gyrus (Khalsa et al., 2009). Finally, Wang et al. (2011) used ASL to investigate perfusion differences between baseline and two yoga/meditation states in Kundalini meditators (*n* = 10). In this study, the different meditation states (a “*focused-based*” practice and a “*breath-based*” practice) were compared to a pre-meditation and post-meditation baseline state. During the focused-based meditation task, increased CBF was observed in the medial frontal gyrus and caudate nucleus, while decreased CBF was detected in the inferior and superior occipital gyrus and inferior parietal lobule compared to the pre-meditation baseline control condition. Several cortical and limbic brain structures showed significant increases in CBF during the breath-based meditation task compared to the pre-meditation control state, including insula, amygdala, hippocampus, parahippocampus, and superior temporal gyrus, indicating different CBF patterns between the two meditation states. Moreover, strong positive correlations were observed between reported depth of meditation and increased CBF responses in the insula, inferior frontal cortex, and temporal pole during the second meditation task, which had positive associations with subjective ratings of both connectedness and depth of meditation as well as a negative association with perceived stress (Wang et al., 2011).

#### Effects of Yoga on Neural Activation

Several fMRI studies investigated brain activations or fluctuations in yoga practitioners (Table 2B and Figure 1C).

Four studies acquired fMRI data during yoga meditation or breathing exercises. Firstly, in a cross-sectional fMRI study performed on eight Kundalini yoga meditation subjects (with less than two years of meditation practice), Engström et al. (2010) investigated whether moderately experienced yoga subjects would display activation in specific brain regions including the hippocampal formation and PFC with an on–off design (meditate vs. word condition). Engström et al. (2010) found activation in different regions when the subjects were meditating using a mantra, compared to pronouncing neutral words (control condition). The most significant activation during the meditation task was found in the bilateral hippocampus/parahippocampal formation. Other areas with significant neuronal activation were found in the middle cingulate cortex and the precentral cortex (Engström et al., 2010). Secondly, changes in activity in the hippocampal formation were also found in a somewhat atypical yoga study where the hemodynamic correlates of “*OM*” chanting were investigated. Healthy participants (*n* = 12, only four of these had formal training in yoga including meditation) were trained in chanting “*OM*” and were scanned during this activation condition (Kalyani et al., 2011). The continuous production of “*ssss*…” was used as control condition. *A priori* ROIs included the limbic brain regions, orbitofrontal cortex, anterior cingulate cortex, and thalami, based on the expected deactivation of these regions due to the vagal effect of the “OM” chanting. Significant deactivation was observed during “*OM*” chanting in the amygdala, anterior cingulate gyrus, hippocampus, insula, orbitofrontal cortex, parahippocampal gyrus, and thalamus (Kalyani et al., 2011). Thirdly, Hernández et al. (2015) investigated increased brain activation during Sahaja yoga meditation. In the control condition, meditators focused their attention on breathing, breathing movements, not worrying, and being relaxed, which is known as mindfulness meditation. After exploring the neural activity pattern with fMRI during yoga meditation (*n* = 19), they found an increased neural activity compared to the control condition in the medial frontal gyrus, anterior cingulate gyrus, and inferior frontal gyrus, insula, superior temporal gyrus, and medial temporal gyrus. The extent of activation relative to the control condition appeared to diminish progressively with later and deeper meditation stages. The authors suggested that this may be due to more focused activation. Additionally, the authors reported that the activation in the temporal lobes may be due to the presence of music during the meditation condition, in contrast to its absence during the control relaxation condition (Hernández et al., 2015). Fourthly, in parallel with the findings of Hernández et al. (2015), increased activation in the right prefrontal regions during a meditation phase (auditory fixation and visual fixation) was observed in four experienced *Patanjali* yoga practitioners. The authors did not report any statistics, detailed demographics, or scan acquisition parameters (Mishra et al., 2017).

Only one study examined neuronal activity during resting state in a population of females diagnosed with schizophrenia spectrum, comparing a yoga intervention (*n* = 23), with aerobic exercises (*n* = 23) and a control wait-list (*n* = 12) group (Lin et al., 2017). Local spontaneous neuronal fluctuations at rest, measured by the amplitude of low-frequency fluctuations (ALFF), have previously shown alterations in patients with schizophrenia (Hoptman et al., 2010). Although no results were reported regarding the differences at baseline between the groups, the yoga group exhibited significantly different ALFF in the precuneus and visual cortex compared to the control group and aerobic exercises group. Furthermore, the yoga intervention induced a significant decrease in ALFF in the precuneus (Lin et al., 2017).

Finally, neuronal activity during an emotional evoking or working memory task in experienced yoga practitioners were investigated by three different groups. More specifically, Wadden et al. (2018) investigated the effects on emotional regulation in experienced yoga subjects (*n* = 19, yoga styles unknown) compared to recreational athletes (*n* = 12, group-sports) using emotionally arousing visual stimuli. A higher activation during the emotion-evoking phase was found in the superior parietal lobule, postcentral gyrus, and anterior supramarginal gyrus of yoga subjects. These areas have been associated with attentional awareness and reduced egocentric bias. However, recreational athletes exhibited higher activation during the emotion-evoking phase in the inferior frontal gyrus and lateral occipital cortex, regions linked with cognitive reappraisal during emotional regulation (Wadden et al., 2018). Similarly, the neurocognitive correlates of emotion interference on cognition were examined in an fMRI study performed by Froeliger et al. (2012a). For this study, yoga practitioners (*n* = 7) and an age-, sex-, and years of education-matched control group (*n* = 7) had to perform an event-related affective viewing trial and Stroop trial during the fMRI session, preceded by a negative or neutral emotional distractor. During the viewing trials, greater activity was observed in the dorsolateral PFC. In contrast, for the Stroop trial, greater activation was observed in the superior frontal gyrus due to the group effect, where the control group showed larger activation responses. An interaction effect of the Stroop trial was seen in the ventrolateral PFC. Froeliger et al. (2012a) suggested that yoga meditation practitioners may selectively recruit dissociable frontal executive-dependent strategies in response to emotionally salient information. Only one study investigated the effects of a long-term yoga practice (consisting of meditation, physical postures, and breathing exercises) on working memory, performing a Sternberg working memory task during fMRI (Gothe et al., 2018). Twenty-six subjects whereof 13 experienced yoga practitioners and 13 age- and sex-matched controls were included in this study. Although no significant difference was found in terms of reaction time and performance accuracy between both groups, the fMRI results showed less activation in the dorsolateral PFC in yoga practitioners during the encoding phase of the Sternberg task. This result may point toward increased efficiency by experienced yoga practitioners while performing the task, which is in line with behavioral studies suggesting the positive influence of yoga on working memory performance (Gothe et al., 2018).

#### Effects of Yoga on Functional Connectivity

Besides differences in brain activation, fMRI has also been used to investigate the potential effect of yoga on functional connectivity in the brain (Table 2C and Figure 1D). Methods used to investigate functional connectivity in the brain include ROI-to-ROI analyses, seed-to-voxel analyses, independent component analyses, and network-based statistics. Within the included studies, the following resting-state networks (RSNs) were investigated: DMN, dorsal attention network (DAN), executive control network (ECN), salience network, language network, and superior parietal network. For the seed basis analyses, most of the seeds were placed in the DMN, including the medial prefrontal cortex (mPFC) and precuneus/posterior cingulate cortex (PCC).

Froeliger et al. (2012a) investigated the effects of Hatha yoga meditation and meditation-state on four RSNs (DMN, DAN, ECN, and salience network) in a ROI-to-ROI analysis, hypothesizing that yoga meditation would be associated with greater functional connectivity between multiple RSNs. Firstly, higher interregional correlation values for the DAN in yoga practitioners (*n* = 7) compared to the controls (*n* = 7) were found. Secondly, interregional connectivity within nodes of the DAN was significantly higher for the yoga group. Within the yoga group, greater functional connectivity was observed during the meditative state between DAN and DMN nodes and between the anterior prefrontal node of the salience network and multiple DAN nodes, compared to the resting state. In contrast, during resting state, greater functional connectivity was found between the DAN and dorso mPFC (node of the ECN) and right insula (node of the salience network), compared to the meditative state (Froeliger et al., 2012a). In a seed-to-voxel fMRI study, Hernández et al. (2016) performed a functional connectivity analysis in the same subjects as their VBM study. They found a positive correlation between subjective perception of the depth of mental silence and GM density in mPFC. This region was subsequently used as a seed region for the seed-to-voxel functional connectivity analyses (Hernández et al., 2018). During the meditation state, functional connectivity between mPFC (seed/ROI) and anterior insula/putamen bilaterally increased significantly. In contrast, functional connectivity was significantly decreased between the mPFC and thalamus/parahippocampal gyrus throughout both the meditation and resting state (Hernández et al., 2018). Similarly, Sevinc et al. (2018) also used the mPFC as seed region to evaluate the common and dissociable neural correlates of an MBSR (*n* = 18) intervention (including yoga poses) and a relaxation response (RR; *n* = 16) program in a longitudinal setting. For this study, two *a priori* seeds were used: the mPFC (associated with focused attention) and the anterior insula (for its role in somatosensory awareness). Both interventions revealed functional coupling between mPFC (seed) and supplementary motor areas during meditation compared to rest. However, the MBSR program was uniquely associated increased functional connectivity between the anterior insula (seed) and the pregenual anterior cingulate cortex during body scan meditation compared with rest (Sevinc et al., 2018). Santaella et al. (2019) used the same seed (mPFC as main anterior seed of the DMN) to address neuronal connectivity in an healthy elderly female population (*n* = 40; same subjects as in Afonso et al., 2017). They found greater resting-state connectivity between the mPFC and the angular gyrus in the yoga group (*n* = 20), compared to paired yoga-naïve controls (*n* = 20) (Santaella et al., 2019).

In total, two studies used an independent component analysis (ICA) approach to investigate functional connectivity within and between identified networks. In an interventional longitudinal fMRI study, Eyre et al. (2016) investigated the correlation between functional connectivity and performance on memory tests, before and after yoga training (*n* = 14) or MET (*n* = 14) in patients with MCI. Subjects were randomly assigned to both groups. Analysis was performed on four networks identified by the ICA that are relevant in the research regarding long-term memory, including DMN, posterior DMN, language network, and superior parietal network. Results showed that improvement on the verbal memory performance was significantly positively correlated with greater connectivity within the DMN. Additionally, a significant negative correlation between changes in functional connectivity and changes in long-term visuospatial memory performance was observed in a cluster within the superior parietal network in both groups (Eyre et al., 2016). Applying an ICA as well, in addition to the VBM analysis, Dodich et al. (2019) also performed rsfMRI in the same subjects. A significant interaction effect (group × time) was observed in the frontal sector of the fronto-parietal network. Within this network, after Sahaja yoga training, decreased power was displayed at ultra-low frequencies and increased power in low-middle frequencies. This change in the power spectrum in the fronto-parietal network was correlated with well-being scores (Dodich et al., 2019).

Finally, in an attempt to disentangle differences between yoga and meditation as such, Gard et al. (2014) investigated functional connectivity in three different groups: yoga practitioners (*n* = 16), meditation practitioners (*n* = 16), and a control group (*n* = 15). Using Network-Based Statistics (NBS), which detects clusters of connections that significantly differ between groups, significant difference components for the comparison yoga practitioners > controls were found, comprising three nodes, with the caudate nucleus as the central node, connected to the parahippocampal gyrus and inferior temporal gyrus. The comparison meditators vs. controls and yoga vs. meditators did not yield significant results; however, significant higher-degree centrality for both meditators and yoga practitioners was observed compared to controls. Furthermore, compared to controls, meditators and yoga practitioners revealed equally stronger connectivity to a large number of brain regions (Gard et al., 2014).

#### Effects of Yoga on Brain Metabolites and Neurotransmitters

So far, only one PET study investigated endogenous striatal dopamine release during yoga meditation using ^11^C-raclopride PET in experienced yoga Nidra practitioners (*n* = 8), during rest and relaxation meditation on two separate days, in random order (Kjaer et al., 2002). A significant 8% decrease in binding potential was observed during meditation in the ventral striatum, associated with the experience of reduced readiness for action, indicating an increased dopaminergic signaling in the striatal regions most closely associated with reward and pleasurable effects (Kjaer et al., 2002).

In the first MRS study, Streeter et al. (2007) instructed one group of eight experienced yoga participants to practice a yoga session, especially focusing on yoga physical postures, while the control group (*n* = 11) had to read for 60 min before the MRS scan. MRS voxels were placed in the cortex and deep GM structures (exact location not specified). In yoga practitioners, an increase in GABA levels was found after a yoga practice while no changes were observed in the control subjects after the reading exercise (Streeter et al., 2007). In a subsequent interventional study, the same group measured MRS GABA levels in the left thalamus in healthy subjects that were randomly assigned to a 60-min yoga (*n* = 19) or walking (*n* = 15) intervention, three times a week for 12 weeks (Streeter et al., 2010). The yoga subjects reported a greater improvement in mood and anxiety, compared to the walking group. Although no significant changes in thalamic GABA levels between groups were found, a significant positive correlation between changes in mood scales (revitalization, tranquility, state-trait anxiety trait) and changes in thalamic GABA levels was found in the yoga group (Streeter et al., 2010).

One study investigated the effects of a yoga intervention in patients with type 2 diabetes on N-acetyl aspartate (NAA) and myoinositol (MI) brain metabolites in the right dorsolateral frontal lobe by means of MRS (Nagothu et al., 2015). In this case–control study, a total of 68 patients, were assigned to either a yoga group (*n* = 34) or a control group (*n* = 34). The yoga group did yoga (including physical postures and breathing exercises) for six months, 6 days a week, for 45–60 min under daily supervision of a qualified yoga teacher. The control group was not on any specific exercise regimen. Neither exact dropout numbers nor the exact age and number of females vs. males of each group are reported by Nagothu et al. (2015). Higher NAA and lower MI levels were found in the yoga group compared to the control group in the right dorsolateral frontal lobe, pointing toward higher neuronal integrity and lower neuroglial functioning, respectively. No baseline values were reported (Nagothu et al., 2015). In another MRS study, with the same study design and intervention as the previously mentioned research, no differences in NAA and MI values were found between the control (*n* = 5) and yoga (*n* = 5) groups (Santhakumari et al., 2016).

In addition to VBM effects, Yang et al. (2016) also acquired MRS in 25 MCI patients subjects, randomized to either a yoga group (*n* = 14) or a memory enhancement training group (*n* = 11). The MRS study was conducted with ROIs placed in the hippocampi and dorsal anterior cingulate cortex. A significant interaction effect between time and group was detected for choline and *post hoc* analysis showed that choline decreased in the hippocampi after memory training, but remained unchanged in the yoga group. Of note, at baseline choline levels were greater in the memory training group compared to the yoga group (Yang et al., 2016).

A summary of significant results related to these functional neuroimaging studies in yoga is provided in Tables 2A–D and illustrated in Figure 1B (effects on CBF and glucose metabolism, showing mainly increases in frontal regions and a decrease in posterior regions; however, each yoga meditative content showed a distinctive pattern), Figure 1C (neuronal activation, showing mainly increased activation in frontal regions during yoga and decreases in posterior regions), Figure 1D (functional connectivity, showing increased functional connectivity in mainly prefrontal regions within DMN), and Figure 1E (neurotransmitters and metabolites, showing widespread effects depending on the yoga intervention and measured metabolite).

## Discussion

Although the published studies on neuroimaging effects of yoga have been characterized by various forms of yoga, mostly smaller groups and heterogeneous target assessment, relatively consistent cerebral structural and functional changes have been found. These may be linked to presumed beneficial physical and psychological effects of yoga reported in a growing body of clinical research studies (Field, 2011, 2016; Vancampfort et al., 2012; Villemure et al., 2015).

Overall, morphological neuroimaging findings are consistent with an increase in regional GM density or volume in yoga practitioners compared to controls. Even though the insular cortex was not employed as an ROI in the included studies, higher GM density in the insular cortex was most consistently reported in the whole-brain analyses (Froeliger et al., 2012b; Villemure et al., 2015; Hernández et al., 2016; Figure 1A). These results are congruent with morphometric neuroimaging of meditation practitioners, where it was found that increases in the insular cortex were the most consistent structural alterations across meditation styles (Fox et al., 2014). In addition to morphological effects in the insula, multiple functional studies included in this review (Kalyani et al., 2011; Wang et al., 2011; Froeliger et al., 2012a; Hernández et al., 2015, 2018) showed differences in the activation or connectivity of the insula in yoga practitioners (Figures 1C,D). Importantly, many of these did not investigate the full triad of yoga but only included yoga styles focusing on meditation without physical postures (*asana*). In parallel, a meta-analysis and systematic review on the functional effects of meditation, including mindfulness-based interventions, reported the consistent recruitment of the insula across multiple styles of meditation (Fox et al., 2016; Young et al., 2018), suggesting an important role for the insula across meditative processes. The insula is central in interoceptive body awareness, the sensitivity toward the psychological conditions of one’s own body (e.g., sensing increased heart rate, sweaty skin, tense stomach, etc.), and empathy and possibly even metacognition (Gu et al., 2012; Simmons et al., 2013). The insula is involved during meditation but also upon postural changes and slow breathing (Critchley et al., 2015). Various neuroimaging studies have shown that higher interoceptive awareness is associated with increased ability of effective stress coping, whereas low interoceptive awareness has been observed in patients with depression (Avery et al., 2014). Yoga may induce an increase in functional connectivity between the insula and brain regions involved in regulation of affective and sensory processing (such as the PFC), which may induce higher GM density in the insular cortex.

Furthermore, increased GM volumes or density in the hippocampus were consistently observed in different morphological studies (Froeliger et al., 2012b; Hariprasad et al., 2013; Villemure et al., 2015; Gothe et al., 2018), including yoga studies containing the full triad of yoga. In line with these results, several functional and structural neuroimaging studies of meditation also reported differences in the hippocampal formation (Fox et al., 2014, 2016). These effects could be linked to increased BDNF levels, a marker for neuroplasticity, as observed after a yoga intervention (Naveen et al., 2016). BDNF may play a role in the morphological effects observed in the hippocampus as it is found in high concentrations within this region (Murer et al., 2001). In contrast, in a large population study in yoga and meditation practitioners (without making a distinction between both), decreased hippocampal volumes were observed at a cross-sectional level, compared to controls, which might be attributed to early life stressors. Practicing meditation and yoga had no significant relation over time with hippocampal volume (Gotink et al., 2018).

The amygdala was explicitly investigated in two morphological structural studies, where decreases in density and volume were observed (Hölzel et al., 2009; Gotink et al., 2018). A significant correlation was found between changes in perceived stress scores and the amygdala volume (Hölzel et al., 2009). Indeed, extensive evidence showed neural correlates of stress-induced modulation of structure and function of both amygdala and hippocampus. It therefore seems plausible that the smaller amygdala and increased hippocampal volumes are due to less experienced stress through yoga practice (Roozendaal et al., 2009; McEwen et al., 2016; McEwen, 2017; Gotink et al., 2018).

Functional neuroimaging studies investigating differences in neural activation during yoga meditation, in comparison to an emotional task or cognitive functioning, found yoga-induced effects in various cortical and subcortical brain regions. However, heterogeneous results across all studies were obtained, which may be due to the heterogeneous nature of the study design, as well as multiple yoga styles and tasks during the functional activity imaging acquisition.

From the five studies focusing on CBF/perfusion and glucose metabolism changes during a yoga meditation practice, it was shown that CBF is influenced by yoga meditation (Herzog et al., 1991; Lou et al., 1999; Cohen et al., 2009; Khalsa et al., 2009; Wang et al., 2011), but influences on CBF may depend heavily on the type and focus of the meditation (Fox et al., 2016; Figure 1B). Eight studies investigated neural activation changes during yoga meditation, with a majority of the studies showing increased activation in the frontal regions during yoga meditation (Froeliger et al., 2012c; Hernández et al., 2015; Lin et al., 2017; Mishra et al., 2017). Functional connectivity studies (Froeliger et al., 2012a; Gard et al., 2015; Eyre et al., 2016; Hernández et al., 2018; Sevinc et al., 2018; Dodich et al., 2019; Santaella et al., 2019) observed improved connectivity in yoga subjects, compared to controls, in multiple networks and regions. Half of the studies on functional connectivity focused on the DMN, using seed-based analyses with seeds in the PFC (Hernández et al., 2018; Sevinc et al., 2018; Santaella et al., 2019). Studies investigated different yoga styles, and heterogeneous results were found, but with a consistent increased connectivity toward the PFC. In general, neural activity within the DMN is known to correlate with mind-wandering, which is in return associated with lower levels of happiness. A possible way to reduce mind-wandering is yoga meditation. Meditation has previously been linked to stronger coupling between the DMN and regions implicated in self-monitoring and cognitive control (Brewer et al., 2011).

Only one study investigated the effect of yoga (*yoga nidra meditation*) on dopaminergic function using the dopamine D_2__/__3_ receptor radioligand ^11^C-raclopride, demonstrating a significant increased dopaminergic release during the Yoga Nidra meditation practice in the ventral striatum (Kjaer et al., 2002). The ventral striatum plays an important role in the circuitry underlying goal-directed behaviors, behavioral sensitization, and changes in affective states (Ito et al., 2004), as well as in the reward/motivation circuitry (Volkow et al., 2012). It should also be noted that the dopaminergic system, via the basal ganglia, participates in regulating the subcortical-prefrontal interactions. In total, five studies used MR spectroscopy to investigate changes in metabolite concentrations in the brain. Nearly all studies employed different ROIs. Increased GABA levels, associated with stress reduction, decreased MI, and increased NAA levels have been reported after yoga (Streeter et al., 2007, 2010; Nagothu et al., 2015). Increased GABA levels might be the result of the activated PFC during or after yoga, as observed in multiple yoga studies (Froeliger et al., 2012c; Hernández et al., 2015; Lin et al., 2017; Mishra et al., 2017), which is believed to increase glutamate, stimulating the reticular nucleus of the thalamus to increase secretions of GABA (Guglietti et al., 2013).

A postulated neurobiological model comprising underlying mechanisms on central yoga effects is that yoga induces increases in CBF, cerebral activity, and glucose metabolism, reflecting recruitment of additional higher demand of cortical substrates, while decreases suggest that the yoga practice can be carried out using fewer neuronal resources as learning proceeds (Dayan and Cohen, 2011). Repeated functional activation might induce morphological changes affecting white and GM through dendritic arborization/synaptogenesis, neurogenesis (limited to the hippocampus), myelin remodeling, and fiber reorganization (Dayan and Cohen, 2011; Zatorre et al., 2012; Lövdén et al., 2013). In return, changes in functional connectivity can occur. Furthermore, multiple bottom-up (e.g., controlled breathing and physical postures) and top-down (e.g., focused attention and interoception) processes also play an important role in mind–body interventions such as yoga (Muehsam et al., 2017). The beneficial effects of yoga practice is that the combination of its three main components—deep breathing, meditation, and physical postures—can activate the parasympathetic nervous system and thereby increase the GABA concentration, the main inhibitory neurotransmitter in the brain, consistent with the findings of Streeter et al. (2007, 2010). An augmenting effect of parasympathetic tone can occur through vagal nerve afferents caused by the baroreflex response that is mainly elicited by the deep and slow breathing, and by activated baroreceptors through different yoga poses (chin lock, inversion, and chest opening poses) during a yoga session (Streeter et al., 2012). This may also be a basis for the anxiety- and stress-relieving effects of yoga. Counteracting stress-related corticosteroid release reduces GABA, which can lead to neurological morphological changes that could have long-term consequences (Roozendaal et al., 2009; McEwen et al., 2016).

In this review, we attempted to summarize the application of neuroimaging (including MRI, PET, and SPECT) to study the neurobiological effects of yoga. Nevertheless, a formal meta-analysis of the results was not considered appropriate because of the relatively small and heterogeneous number of consistently designed studies. These relatively small sample sizes may also have limited the sensitivity to identify effects. Additionally, 38% of the studies (13/34) did not include a control group. The selection of a control group and control conditions is quite heterogeneous and varies from cognitive to motor task, making it difficult to compare studies. Physical exercise, or matching based on physical exercise level, was in general most consistently used as control group or condition, respectively. This is in line with the results of a systematic review regarding comparison groups in yoga research (Park et al., 2014). Physical exercise studies allow to establish whether the yoga effect is mainly determined by exercise or whether yoga provides an additional effect. Given its importance in order to control for non-specific effects of group participation, attention, and activity (Park et al., 2014) but scarcity of studies using an active control group, we strongly advocate incorporation in future study designs. In studies on effects of physical activity on brain structure and function, several similarities have been found. In general, physical activity (or aerobic training) has also been associated with increased GM density. Areas that were most consistently observed included the hippocampus, PFC, and motor-related areas such as cerebellum and motor cortex (for a review, see Thomas et al., 2012 and Erickson et al., 2014). In addition, a review of Sexton et al. showed that a relation between physical activity and WM structure has been suggested in multiple studies (Sexton et al., 2016). Regarding functional activity changes, Herold et al. (2020) reported profound changes especially in the frontal lobe, cerebellum, and hippocampus in response to exercise. Therefore, especially regarding cognitive, executive, and motor effects that can be associated with yoga practice, future study designs should clearly try to demonstrate whether structural and functional effects are different from a pure physical exercise component. This is a complex effort, as also physical exercise can be modulated by many variables, including exercise intensity, duration, aerobic vs. anaerobic, and a range of regional or global exercise levels.

Similarly, variations in style and intensity of yoga interventions were present in the reviewed studies, which makes it difficult to find clear common ground for the findings. Moreover, half of the yoga-related neuroimaging studies included in this review investigated the effects of yoga styles that mainly focus on meditation, whereas the other half investigated yoga including physical postures (*asana*). The latter is the type of yoga that is mostly practiced in the Western world nowadays, with even a primary focus on the physical postures. The different yoga styles may have different effects on the brain, since each yoga style differentially emphasizes the subcomponents of yoga. The study by Villemure et al. (2015) indicated that postures, breathing exercises, and meditation contribute differently to the structural changes observed after a series of yoga practices (Villemure et al., 2015). About three-fourths of the study subjects were female; however, this in line with the observations that lifetime yoga practitioners are more likely female (Cramer et al., 2016). Most studies did not use randomization. Four studies were of a mere exploratory design and did not control for multiple comparisons (Lou et al., 1999; Cohen et al., 2009; Hariprasad et al., 2013; Hernández et al., 2016), increasing the chance of false-positive findings.

Future research should be aimed at disentangling the effects of each yoga subcomponent on specific neuronal patterns and the interaction between these subcomponents, in larger and better-defined populations. Finally, the general issue of publication bias is likely also present, where negative findings may remain unreported (Egger et al., 1997).

## Conclusion

Based on the relatively scarce but expanding neuroimaging evidence of yoga practice in predominantly healthy subjects, it has been shown that yoga has both a structural and functional effect on brain areas involved in interoception, posture, motivation, and higher executive functions. Overall, most consistent structural effects were observed in the hippocampus and insular cortex, while functional studies showed mainly increases in frontal executive and attention areas. However, the number of studies is still limited and heterogeneous and several inconsistencies are present due to the heterogeneity among the different yoga styles included and the great variability in the applied research protocols.

More extensive, well-designed, and multimodal/multiparametric research studies with the control group preferably including physical exercise should be performed to further investigate the potential beneficial effects of yoga not exclusively on the healthy brain but also in disease state, for example in mood and anxiety disorders such as major depression, PTSD, or anxiety states. The integration of both neuroimaging and neurophysiological techniques (EEG, EMG, etc.) will further allow to investigate and bridge imaging findings with neurophysiological and behavioral assessment/improvements in well-being.

## Author Contributions

JvA performed the PubMed search and wrote the review. JC and KVL wrote the review and critically revised the manuscript for intellectual content. KD and SS critically revised the manuscript for intellectual content. All authors contributed to the article and approved the submitted version.

## Conflict of Interest

The authors declare that the research was conducted in the absence of any commercial or financial relationships that could be construed as a potential conflict of interest.

## Footnotes

1 http://www.ncbi.nlm.nih.gov/pubmed/

## References

[B1] Afonso R. F. Balardin J. B. Lazar S. Sato J. R. Igarashi N. Santaella D. F. (2017). Greater cortical thickness in elderly female yoga practitioners-a cross-sectional study. *Front. Aging Neurosci.* 9:201. 10.3389/fnagi.2017.00201 28676757 PMC5476728

[B2] Avery J. A. Drevets W. C. Moseman S. E. Bodurka J. Barcalow J. C. Simmons W. K. (2014). Major depressive disorder is associated with abnormal interoceptive activity and functional connectivity in the insula. *Biol. Psychiatry* 76 258–266. 10.1016/j.biopsych.2013.11.027 24387823 PMC4048794

[B3] Barnes P. M. Bloom B. Nahin R. L. (2008). *Complementary and Alternative Medicine use Among Adults and Children: United States, 2007.* Hyattsville, MD: National Center for Health Statistics, 1–23. 19361005

[B4] Birdee G. S. Sohl S. J. Wallston K. (2016). Development and psychometric properties of the yoga self-efficacy scale (YSES). *BMC Complement. Alternat. Med.* 16:3. 10.1186/s12906-015-0981-0 26738919 PMC4704262

[B5] Brenes G. A. Sohl S. Wells R. E. Befus D. Campos C. L. Danhauer S. C. (2018). The effects of yoga on patients with mild cognitive impairment and dementia: a scoping review. *Am. J. Geriatr. Psychiatry* 27 188–197. 10.1016/j.jagp.2018.10.013 30413292 PMC6541218

[B6] Brewer J. A. Worhunsky P. D. Gray J. R. Tang Y.-Y. Weber J. Kober H. (2011). Meditation experience is associated with differences in default mode network activity and connectivity. *Proc. Natl. Acad. Sci. U.S.A.* 108 20254–20259. 10.1073/pnas.1112029108 22114193 PMC3250176

[B7] Catana C. Guimaraes A. R. Rosen B. R. (2013). PET and MR imaging: the odd couple or a match made in heaven? *J. Nucl. Med.* 54 815–824. 10.2967/jnumed.112.112771 23492887 PMC3801211

[B8] Chen J. E. Glover G. H. (2015). Functional magnetic resonance imaging methods. *Neuropsychol. Rev.* 25 289–313. 10.1007/s11065-015-9294-9 26248581 PMC4565730

[B9] Chu P. Gotink R. A. Yeh G. Y. Goldie S. J. Hunink M. M. (2016). The effectiveness of yoga in modifying risk factors for cardiovascular disease and metabolic syndrome: a systematic review and meta-analysis of randomized controlled trials. *Eur. J. Prev. Cardiol.* 23 291–307. 10.1177/2047487314562741 25510863

[B10] Clarke T. C. Barnes P. M. Black L. I. Stussman B. J. Nahin R. L. (2018). Use of yoga, meditation, and chiropractors among U.S. adults aged 18 and over. *NCHS Data Brief* 325 1–8. 30475686

[B11] Cohen D. L. Wintering N. Tolles V. Townsend R. R. Farrar J. T. Galantino M. L. (2009). Cerebral blood flow effects of yoga training: preliminary evaluation of 4 cases. *J. Altern. Complement. Med.* 15 9–14. 10.1089/acm.2008.0008 19769471 PMC3155099

[B12] Cramer H. Lauche R. Langhorst J. Dobos G. (2013). Yoga for depression: a systematic review and meta-analysis. *Depress. Anxiety* 30 1068–1083. 10.1002/da.22166 23922209

[B13] Cramer H. Ward L. Steel A. Lauche R. Dobos G. Zhang Y. (2016). Prevalence, patterns, and predictors of yoga use: results of a U.S. Nationally representative survey. *Am. J. Prev. Med.* 50 230–235. 10.1016/J.AMEPRE.2015.07.037 26497261

[B14] Critchley H. D. Nicotra A. Chiesa P. A. Nagai Y. Gray M. A. Minati L. (2015). Slow breathing and hypoxic challenge: cardiorespiratory consequences and their central neural substrates. *PLoS One* 10:e0127082. 10.1371/journal.pone.0127082 25973923 PMC4431729

[B15] Danucalov M. A. D. Kozasa E. H. Ribas K. T. Galduróz J. C. F. Garcia M. C. Verreschi I. T. N. (2013). A yoga and compassion meditation program reduces stress in familial caregivers of Alzheimer’s disease patients. *Evid. Based Complement. Alternat. Med.* 2013:513149. 10.1155/2013/513149 23690846 PMC3652205

[B16] Dayan E. Cohen L. G. (2011). Neuroplasticity subserving motor skill learning. *Neuron* 72 443–454. 10.1016/j.neuron.2011.10.008 22078504 PMC3217208

[B17] Dodich A. Zollo M. Crespi C. Cappa S. F. Laureiro Martinez D. Falini A. (2019). Short-term Sahaja yoga meditation training modulates brain structure and spontaneous activity in the executive control network. *Brain Behav.* 9:e01159. 10.1002/brb3.1159 30485713 PMC6346416

[B18] Egger M. Davey Smith G. Schneider M. Minder C. (1997). Bias in meta-analysis detected by a simple, graphical test. *BMJ* 315 629–634. 10.1136/BMJ.315.7109.629 9310563 PMC2127453

[B19] Engström M. Pihlsgård J. Lundberg P. Söderfeldt B. (2010). Functional magnetic resonance imaging of hippocampal activation during silent mantra meditation. *J. Alternat. Complement. Med.* 16 1253–1258. 10.1089/acm.2009.0706 21138386

[B20] Erickson K. I. Leckie R. L. Weinstein A. M. (2014). Physical activity, fitness, and gray matter volume. *Neurobiol. Aging* 35 S20–S28. 10.1016/j.neurobiolaging.2014.03.034 24952993 PMC4094356

[B21] Eyre H. A. Acevedo B. Yang H. Siddarth P. Van Dyk K. Ercoli L. (2016). Changes in neural connectivity and memory following a yoga intervention for older adults: a pilot study. *J. Alzheimers Dis.* 52 673–684. 10.3233/JAD-150653 27060939 PMC4927889

[B22] Field T. (2011). Yoga clinical research review. *Complement. Ther. Clin. Pract.* 17 1–8. 10.1016/j.ctcp.2010.09.007 21168106

[B23] Field T. (2016). Yoga research review. *Complement. Ther. Clin. Pract.* 24 145–161. 10.1016/j.ctcp.2016.06.005 27502816

[B24] Fox K. C. R. Dixon M. L. Nijeboer S. Girn M. Floman J. L. Lifshitz M. (2016). Functional neuroanatomy of meditation: a review and meta-analysis of 78 functional neuroimaging investigations. *Neurosci. Biobehav. Rev.* 65 208–228. 10.1016/j.neubiorev.2016.03.021 27032724

[B25] Fox K. C. R. Nijeboer S. Dixon M. L. Floman J. L. Ellamil M. Rumak S. P. (2014). Is meditation associated with altered brain structure? A systematic review and meta-analysis of morphometric neuroimaging in meditation practitioners. *Neurosci. Biobehav. Rev.* 43 48–73. 10.1016/j.neubiorev.2014.03.016 24705269

[B26] Fox M. D. Greicius M. (2010). Clinical applications of resting state functional connectivity. *Front. Syst. Neurosci.* 4:19. 10.3389/fnsys.2010.00019 20592951 PMC2893721

[B27] Froeliger B. Garland E. L. Kozink R. V. Modlin L. A. Chen N. K. McClernon F. J. (2012a). Meditation-state functional connectivity (msFC): strengthening of the dorsal attention network and beyond. *Evid. Based Complement. Alternat. Med.* 2012:680407. 10.1155/2012/680407 22536289 PMC3320106

[B28] Froeliger B. Garland E. L. McClernon F. J. (2012b). Yoga meditation practitioners exhibit greater gray matter volume and fewer reported cognitive failures: results of a preliminary voxel-based morphometric analysis. *Evid. Based Complement. Alternat. Med.* 2012 1–8. 10.1155/2012/821307 23304217 PMC3525089

[B29] Froeliger B. Garland E. L. Modlin L. A. McClernon F. J. (2012c). Neurocognitive correlates of the effects of yoga meditation practice on emotion and cognition: a pilot study. *Front. Integr. Neurosci.* 6:48. 10.3389/fnint.2012.00048 22855674 PMC3405281

[B30] Gard T. Hölzel B. K. Lazar S. W. (2014). The potential effects of meditation on age-related cognitive decline: a systematic review. *Ann. N. Y. Acad. Sci.* 1307 89–103. 10.1111/nyas.12348 24571182 PMC4024457

[B31] Gard T. Taquet M. Dixit R. Hölzel B. K. Dickerson B. C. Lazar S. W. (2015). Greater widespread functional connectivity of the caudate in older adults who practice kripalu yoga and vipassana meditation than in controls. *Front. Hum. Neurosci.* 9:137. 10.3389/fnhum.2015.00137 25852521 PMC4360708

[B32] Goffin K. van Laere K. (2016). Single-photon emission tomography. *Handb. Clin. Neurol.* 135 241–250. 10.1016/B978-0-444-53485-9.00013-1 27432669

[B33] Gothe N. P. Hayes J. M. Temali C. Damoiseaux J. S. (2018). Differences in brain structure and function among yoga practitioners and controls. *Front. Integr. Neurosci.* 12:26. 10.3389/fnint.2018.00026 29988397 PMC6023989

[B34] Gotink R. A. Vernooij M. W. Ikram M. A. Niessen W. J. Krestin G. P. Hofman A. (2018). Meditation and yoga practice are associated with smaller right amygdala volume: the Rotterdam study. *Brain Imaging Behav.* 12 1631–1639. 10.1007/s11682-018-9826-z 29417491 PMC6302143

[B35] Gu X. Gao Z. Wang X. Liu X. Knight R. T. Hof P. R. (2012). Anterior insular cortex is necessary for empathetic pain perception. *Brain* 135 2726–2735. 10.1093/brain/aws199 22961548 PMC3437027

[B36] Guglietti C. L. Daskalakis Z. J. Radhu N. Fitzgerald P. B. Ritvo P. (2013). Meditation-related increases in GABAB modulated cortical inhibition. *Brain Stimul.* 6 397–402. 10.1016/j.brs.2012.08.005 23022436

[B37] Hagins M. Rundle A. Consedine N. S. Khalsa S. B. S. (2014). A randomized controlled trial comparing the effects of yoga with an active control on ambulatory blood pressure in individuals with prehypertension and stage 1 hypertension. *J. Clin. Hypertens.* 16 54–62. 10.1111/jch.12244 24387700 PMC3948002

[B38] Haller S. Zaharchuk G. Thomas D. L. Lovblad K.-O. Barkhof F. Golay X. (2016). Arterial spin labeling perfusion of the brain: emerging clinical applications. *Radiology* 281 337–356. 10.1148/radiol.2016150789 27755938

[B39] Hariprasad V. R. Varambally S. Shivakumar V. Kalmady S. V. Venkatasubramanian G. Gangadhar B. N. (2013). Yoga increases the volume of the hippocampus in elderly subjects. *Indian J. Psychiatry* 55 S394–S396. 24049206 10.4103/0019-5545.116309 PMC3768219

[B40] Hendee W. R. Morgan C. J. (1984). Magnetic resonance imaging. Part I – physical principles. *West J. Med.* 141 491–500. 6506686 PMC1021860

[B41] Henning A. (2018). Proton and multinuclear magnetic resonance spectroscopy in the human brain at ultra-high field strength: a review. *Neuroimage* 168 181–198. 10.1016/j.neuroimage.2017.07.017 28712992

[B42] Hernández S. E. Barros-Loscertales A. Xiao Y. González-Mora J. L. Rubia K. (2018). Gray matter and functional connectivity in anterior cingulate cortex are associated with the state of mental silence during Sahaja yoga meditation. *Neuroscience* 371 395–406. 10.1016/j.neuroscience.2017.12.017 29275207

[B43] Hernández S. E. Suero J. Barros A. González-Mora J. L. Rubia K. (2016). Increased grey matter associated with long-term Sahaja yoga meditation: a voxel-based morphometry study. *PLoS One* 11:e0150757. 10.1371/journal.pone.0150757 26938433 PMC4777419

[B44] Hernández S. E. Suero J. Rubia K. González-Mora J. L. (2015). Monitoring the neural activity of the state of mental silence while practicing Sahaja yoga meditation. *J. Alternat. Complement. Med.* 21 175–179. 10.1089/acm.2013.0450 25671603

[B45] Herold F. Aye N. Lehmann N. Taubert M. Müller N. G. (2020). The contribution of functional magnetic resonance imaging to the understanding of the effects of acute physical exercise on cognition. *Brain Sci.* 10:175. 10.3390/brainsci10030175 32197357 PMC7139910

[B46] Herzog H. Lele V. R. Kuwert T. Langen K.-J. Kops E. R. Feinendegen L. E. (1991). Changed pattern of regional glucose metabolism during yoga meditative relaxation. *Neuropsychobiology* 23 182–187. 10.1159/000119450 2130287

[B47] Hölzel B. K. Carmody J. Evans K. C. Hoge E. A. Dusek J. A. Morgan L. (2009). Stress reduction correlates with structural changes in the amygdala. *Soc. Cogn. Affect. Neurosci.* 5 11–17. 10.1093/scan/nsp034 19776221 PMC2840837

[B48] Hoptman M. J. Zuo X. N. Butler P. D. Javitt D. C. D’Angelo D. Mauro C. J. (2010). Amplitude of low-frequency oscillations in schizophrenia: a resting state fMRI study. *Schizophr. Res.* 117 13–20. 10.1016/j.schres.2009.09.030 19854028 PMC2822110

[B49] Hutton C. Draganski B. Ashburner J. Weiskopf N. (2009). A comparison between voxel-based cortical thickness and voxel-based morphometry in normal aging. *Neuroimage* 48 371–380. 10.1016/j.neuroimage.2009.06.043 19559801 PMC2741580

[B50] Ito R. Robbins T. W. Everitt B. J. (2004). Differential control over cocaine-seeking behavior by nucleus accumbens core and shell. *Nat. Neurosci.* 7 389–397. 10.1038/nn1217 15034590

[B51] Ivtzan I. Jegatheeswaran S. (2015). The yoga boom in western society: practitioners’ spiritual vs. physical intentions and their impact on psychological wellbeing. *J. Yoga Phys. Ther.* 05 1–7. 10.4172/2157-7595.1000204

[B52] Kalyani B. G. Venkatasubramanian G. Arasappa R. Rao N. P. Kalmady S. V. Behere R. V. (2011). Neurohemodynamic correlates of “OM” chanting: a pilot functional magnetic resonance imaging study. *Int. J. Yoga* 4 3–6. 10.4103/0973-6131.78171 21654968 PMC3099099

[B53] Khalsa D. S. Amen D. Hanks C. Money N. Newberg A. (2009). Cerebral blood flow changes during chanting meditation. *Nucl. Med. Commun.* 30 956–961. 10.1097/MNM.0b013e32832fa26c 19773673

[B54] Khalsa S. B. Cohen L. McCall T. Telles S. (2016). The principles and practice of yoga in health care. *Int. J. Yoga* 11 86–87.

[B55] Kjaer T. W. Bertelsen C. Piccini P. Brooks D. Alving J. Lou H. C. (2002). Increased dopamine tone during meditation-induced change of consciousness. *Cogn. Brain Res.* 13 255–259. 10.1016/s0926-6410(01)00106-9 11958969

[B56] Lin J. Chan S. K. Lee E. H. Chang W. C. Tse M. Su W. W. (2015). Aerobic exercise and yoga improve neurocognitive function in women with early psychosis. *NPJ Schizophr.* 1:15047. 10.1038/npjschz.2015.47 27336050 PMC4849465

[B57] Lin J. Geng X. Lee E. H. Chan S. K. Chang W. C. Hui C. L. (2017). Yoga reduces the brain’s amplitude of low-frequency fluctuations in patients with early psychosis results of a randomized controlled trial. *Schizophr. Res.* 184 141–142. 10.1016/j.schres.2016.11.040 27913158

[B58] Lou H. C. Kjaer T. W. Friberg L. Wildschiodtz G. Holm S. Nowak M. (1999). A15O-H2O PET study of meditation and the resting state of normal consciousness. *Hum. Brain Mapp.* 7 98–105. 10.1002/(SICI)1097-019319997:2<98::AID-HBM3<3.0.CO;2-M 9950067 PMC6873339

[B59] Lövdén M. Wenger E. Mårtensson J. Lindenberger U. Bäckman L. (2013). Structural brain plasticity in adult learning and development. *Neurosci. Biobehav. Rev.* 37 2296–2310. 10.1016/j.neubiorev.2013.02.014 23458777

[B60] Lüsebrink F. Wollrab A. Speck O. (2013). Cortical thickness determination of the human brain using high resolution 3T and 7T MRI data. *Neuroimage* 70 122–131. 10.1016/j.neuroimage.2012.12.016 23261638

[B61] McEwen B. S. (2017). Neurobiological and systemic effects of chronic stress. *Chronic Stress* 1:2470547017692328. 10.1177/2470547017692328 28856337 PMC5573220

[B62] McEwen B. S. Nasca C. Gray J. D. (2016). Stress effects on neuronal structure: hippocampus, amygdala and prefrontal cortex. *Neuropsychopharmacology* 41 3–23. 10.1038/npp.2015.171 26076834 PMC4677120

[B63] Mishra S. Singh S. Moheb N. Khosa S. Trikamji B. (2017). Changes in functional magnetic resonance imaging with Yogic meditation: a pilot study. *AYU An Int. Q. J. Res. Ayurveda* 38 108–112. 10.4103/ayu.AYU_34_17 PMC6153914 30254388

[B64] Muehsam D. Lutgendorf S. Mills P. J. Rickhi B. Chevalier G. Bat N. (2017). The embodied mind: a review on functional genomic and neurological correlates of mind-body therapies. *Neurosci. Biobehav. Rev.* 73 165–181. 10.1016/j.neubiorev.2016.12.027 28017838

[B65] Murer M. Yan Q. Raisman-Vozari R. (2001). Brain-derived neurotrophic factor in the control human brain, and in Alzheimer’s disease and Parkinson’s disease. *Prog. Neurobiol.* 63 71–124. 10.1016/S0301-0082(00)00014-9 11040419

[B66] Nagothu R. S. Indla Y. R. Rajagopalan A. Varma R. (2015). Right dorsolateral frontal lobe n-acetyl aspartate and myoinositol concentration estimation in type 2 diabetes with magnetic resonance spectroscopy. *J. Clin. Diagn. Res.* 9 CC16–CC19. 10.7860/JCDR/2015/14153.6234 26393123 PMC4572954

[B67] Naveen G. H. Varambally S. Thirthalli J. Rao M. Christopher R. Gangadhar B. N. (2016). Serum cortisol and BDNF in patients with major depression–effect of yoga. *Int. Rev. Psychiatry* 28 273–278. 10.1080/09540261.2016.1175419 27174729

[B68] Park C. L. Groessl E. Maiya M. Sarkin A. Eisen S. V. Riley K. (2014). Comparison groups in yoga research: a systematic review and critical evaluation of the literature. *Complement. Ther. Med.* 22 920–929. 10.1016/j.ctim.2014.08.008 25440384 PMC4254537

[B69] Riley K. E. Park C. L. (2015). How does yoga reduce stress? A systematic review of mechanisms of change and guide to future inquiry. *Health Psychol. Rev.* 7199 1–18. 10.1080/17437199.2014.981778 25559560

[B70] Roozendaal B. McEwen B. S. Chattarji S. (2009). Stress, memory and the amygdala. *Nat. Rev. Neurosci.* 10 423–433. 10.1038/nrn2651 19469026

[B71] Santaella D. F. Balardin J. B. Afonso R. F. Giorjiani G. M. Sato J. R. Lacerda S. S. (2019). Greater anteroposterior default mode network functional connectivity in long-term elderly yoga practitioners. *Front. Aging Neurosci.* 11:158. 10.3389/fnagi.2019.00158 31312135 PMC6614333

[B72] Santhakumari R. Reddy I. Y. Archana R. Rajesh P. (2016). Role of yoga in alienating the memory decline and frontal lobe metabolite changes in type 2 diabetes. *Int. J. Res. Ayurveda Pharm.* 7 78–81. 10.7897/2277-4343.07116 27390721 PMC4933304

[B73] Sasson E. Doniger G. M. Pasternak O. Assaf Y. (2010). Structural correlates of memory performance with diffusion tensor imaging. *Neuroimage* 50 1231–1242. 10.1016/j.neuroimage.2009.12.079 20045476

[B74] Sevinc G. Hölzel B. K. Hashmi J. Greenberg J. McCallister A. Treadway M. (2018). Common and dissociable neural activity after mindfulness-based stress reduction and relaxation response programs. *Psychosom. Med.* 80 439–451. 10.1097/PSY.0000000000000590 29642115 PMC5976535

[B75] Sexton C. E. Betts J. F. Demnitz N. Dawes H. Ebmeier K. P. Johansen-Berg H. (2016). A systematic review of MRI studies examining the relationship between physical fitness and activity and the white matter of the ageing brain. *Neuroimage* 131 81–90. 10.1016/j.neuroimage.2015.09.071 26477656 PMC4851455

[B76] Simmons W. K. Avery J. A. Barcalow J. C. Bodurka J. Drevets W. C. Bellgowan P. (2013). Keeping the body in mind: insula functional organization and functional connectivity integrate interoceptive, exteroceptive, and emotional awareness. *Hum. Brain Mapp.* 34 2944–2958. 10.1002/hbm.22113 22696421 PMC6870113

[B77] Sossi V. (2018). Advances in PET methodology. *Int. Rev. Neurobiol.* 141 3–30. 10.1016/bs.irn.2018.07.034 30314600

[B78] Streeter C. C. Gerbarg P. L. Saper R. B. Ciraulo D. A. Brown R. P. (2012). Effects of yoga on the autonomic nervous system, gamma-aminobutyric-acid, and allostasis in epilepsy, depression, and post-traumatic stress disorder. *Med. Hypotheses* 78 571–579. 10.1016/j.mehy.2012.01.021 22365651

[B79] Streeter C. C. Jensen J. E. Perlmutter R. M. Cabral H. J. Tian H. Terhune D. B. (2007). Yoga asana sessions increase brain GABA levels: a pilot study. *J. Alternat. Complement. Med.* 13 419–426. 10.1089/acm.2007.6338 17532734

[B80] Streeter C. C. Whitfield T. H. Owen L. Rein T. Karri S. K. Yakhkind A. (2010). Effects of yoga versus walking on mood, anxiety, and brain GABA levels: a randomized controlled MRS study. *J. Alternat. Complement. Med.* 16 1145–1152. 10.1089/acm.2010.0007 20722471 PMC3111147

[B81] Thomas A. G. Dennis A. Bandettini P. A. Johansen-Berg H. (2012). The effects of aerobic activity on brain structure. *Front. Psychol.* 3:86. 10.3389/fpsyg.2012.00086 22470361 PMC3311131

[B82] Trotta N. Baete K. Van Laere K. Goldman S. De Tiège X. Wens V. (2018). Neurometabolic resting-state networks derived from seed-based functional connectivity analysis. *J. Nucl. Med.* 59 1642–1643. 10.2967/jnumed.118.212878 29700128

[B83] Vandenberghe S. Mikhaylova E. D’Hoe E. Mollet P. Karp J. S. (2016). Recent developments in time-of-flight PET. *EJNMMI Phys.* 3:3. 10.1186/s40658-016-0138-3 26879863 PMC4754240

[B84] Vancampfort D. Vansteelandt K. Scheewe T. Probst M. Knapen J. De Herdt A. (2012). Yoga in schizophrenia: a systematic review of randomised controlled trials. *Acta Psychiatr. Scand.* 126 12–20. 10.1111/j.1600-0447.2012.01865.x 22486714

[B85] Vardar Yağlı N. şener G. Arıkan H. Sağlam M. İnal İnce D. Savcı S. (2015). Do yoga and aerobic exercise training have impact on functional capacity, fatigue, peripheral muscle strength, and quality of life in breast cancer survivors? *Integr. Cancer Ther.* 14 125–132. 10.1177/1534735414565699 25567329

[B86] Verma A. Kumar I. Verma N. Aggarwal P. Ojha R. (2016). Magnetic resonance spectroscopy – revisiting the biochemical and molecular milieu of brain tumors. *BBA Clin.* 5 170–178. 10.1016/j.bbacli.2016.04.002 27158592 PMC4845155

[B87] Villemure C. Ceko M. Cotton V. A. Bushnell M. C. (2014). Insular cortex mediates increased pain tolerance in yoga practitioners. *Cereb. Cortex* 24 2732–2740. 10.1093/cercor/bht124 23696275 PMC4153807

[B88] Villemure C. Čeko M. Cotton V. A. Bushnell M. C. (2015). Neuroprotective effects of yoga practice: age-, experience-, and frequency-dependent plasticity. *Front. Hum. Neurosci.* 9:281. 10.3389/fnhum.2015.00281 26029093 PMC4428135

[B89] Volkow N. D. Wang G.-J. Tomasi D. Kollins S. H. Wigal T. L. Newcorn J. H. (2012). Methylphenidate-elicited dopamine increases in ventral striatum are associated with long-term symptom improvement in adults with attention deficit hyperactivity disorder. *J. Neurosci.* 32 841–849. 10.1523/JNEUROSCI.4461-11.2012 22262882 PMC3350870

[B90] Wadden K. P. Snow N. J. Sande P. Slawson S. Waller T. Boyd L. A. (2018). Yoga practitioners uniquely activate the superior parietal lobule and supramarginal gyrus during emotion regulation. *Front. Integr. Neurosci.* 12:60. 10.3389/fnint.2018.00060 30564105 PMC6289073

[B91] Wang D. J. J. Rao H. Korczykowski M. Wintering N. Pluta J. Singh D. (2011). Cerebral blood flow changes associated with different meditation practices and perceived depth of meditation. *Psychiatry Res. Neuroimaging* 191 60–67. 10.1016/j.pscychresns.2010.09.011 21145215

[B92] Wieland L. S. Skoetz N. Pilkington K. Vempati R. D’Adamo C. R. Berman B. M. (2017). Yoga treatment for chronic non-specific low back pain. *Cochrane Database Syst. Rev.* 1:CD010671. 10.1002/14651858.CD010671.pub2 28076926 PMC5294833

[B93] Woolery A. Myers H. Sternlieb B. Zeltzer L. (2004). A yoga intervention for young adults. *Alternat. Ther. Health Med.* 10 60–63. 15055096

[B94] Yang H. Leaver A. M. Siddarth P. Paholpak P. Ercoli L. St Cyr N. M. (2016). Neurochemical and neuroanatomical plasticity following memory training and yoga interventions in older adults with mild cognitive impairment. *Front. Aging Neurosci.* 8:277. 10.3389/fnagi.2016.00277 27917121 PMC5116460

[B95] Young K. S. van der Velden A. M. Craske M. G. Pallesen K. J. Fjorback L. Roepstorff A. (2018). The impact of mindfulness-based interventions on brain activity: a systematic review of functional magnetic resonance imaging studies. *Neurosci. Biobehav. Rev.* 84 424–433. 10.1016/j.neubiorev.2017.08.003 28797556

[B96] Zatorre R. J. Fields R. D. Johansen-Berg H. (2012). Plasticity in gray and white: neuroimaging changes in brain structure during learning. *Nat. Neurosci.* 15 528–536. 10.1038/nn.3045 22426254 PMC3660656

