# Supplementary material for: What Has Neuroimaging Taught Us on the Neurobiology of Yoga? A Review
Source: Front Integr Neurosci. 2020 Jul 8;14:34. doi: 10.3389/fnint.2020.00034 (PMC7362763; doi:10.3389/fnint.2020.00034)
Supplement: Supplementary file 1 [file Data_Sheet_1.DOCX]

# Supplementary 1

- *Iyengar* yoga, named after one of the well-known yoga masters R.K.S. Iyengar, strives for precise anatomical alignment in poses. Various props like straps, bolsters, blocks, ropes, benches of different sizes and shapes, are used to allow students who lack sufficient flexibility to reach anatomical alignment (McCall, 2007).
- *Ashtanga* yoga, based on the teachings of K. Pattabhi Jois of Mysore in India, is one of the most vigorous styles of yoga. Practitioners perform fixed series of postures, while breathing in the style of *Ujjayi pranayama* (ocean’s breath) (McCall, 2007).
- *Vinyasa (*Sanskrit for “movement of limbs”) yoga includes a vigorous workout with repeated Sun Salutations. However, it is not a rigidly defined set of poses and incorporates variations of the Ashtanga theme. Movement is coordinated with the breath to flow from one pose to the next (McCall, 2007).
- *Kripalu* yoga emphasizes on creating an emotionally safe space to practice and go into and “process” feelings, reflecting Kripalu’s conscious integration of insights from Western psychotherapy (McCall, 2007).
- *Kundalini* yoga incorporates movement, dynamic breathing techniques, meditation, and the chanting of mantras. The goal is to build physical vitality and increase consciousness that activates energy centres throughout the body (McCall, 2007).
- Yoga *Nidra* (Sanskrit for yogic “sleep"), is a form of guided relaxation. While lying down in *savasana* (corpse pose), the practitioners are taken on a journey through dozens of different visualizations. A teacher or a recorded voice guides the practitioners through the practice. The mind stays occupied, by keeping the attention to the voice and different areas of the body (McCall, 2007).
- *Sahaja* yoga is a unique method of meditation based on an experience called Self Realization that can occur within each human being and aims to achieve a state of mental silence. Through this process, an inner transformation takes place by which one becomes moral, united and balanced (Elías Hernández et al., 2016).
- *Sivananda* yoga, named after *Sivananda Saraswati*, focuses on preserving health and wellness and is based on five principles: proper exercise (*asana*), proper breathing (*pranayama*), proper relaxation (*savasana*), proper diet (vegetarian), positive thinking (*vendata*) and meditation (*dyana*) (Vishnu Devananda, 1955).
- *Hatha* yoga is a general category that includes many yoga styles. It integrates physical poses and breathing exercises, which help to bring peace to the mind and body while preparing the body for deeper spiritual practices such as meditation (Froeliger et al., 2012b).
